# Research hotspots and trends of sarcopenia in orthopaedic surgery: a bibliometric analysis from 2003 to 2023

**DOI:** 10.3389/fsurg.2025.1564923

**Published:** 2025-05-06

**Authors:** Ruijiang Li, Yufeng Ge, Yixiao Chen, Gang Liu, Feng Gao, Chao Tu, Ting Li, Ling Wang, Minghui Yang, Xinbao Wu

**Affiliations:** ^1^ Department of Orthopedics and Traumatology, Beijing Jishuitan Hospital, Capital Medical University, Beijing, China; ^2^ National Center for Orthopaedics, Beijing, China; ^3^ Department of Radiology, Beijing Jishuitan Hospital, Capital Medical University, Beijing, China

**Keywords:** sarcopenia, osteoporosis, bibliometric analysis, VOSviewer, CiteSpace

## Abstract

**Purpose:**

This study aimed to evaluate the current bibliometric characteristics, progress, and hotspots of cross-sectional research on orthopaedic surgery and sarcopenia over the past two decades.

**Methods:**

Publications related to sarcopenia and orthopaedic surgery, published between January 2003 and December 2023, were screened the Web of Science Core Collection. The bibliometric analysis and data visualization processes—including assessments of authors, countries, institutions, keywords, and references—were conducted with Microsoft Office Excel, VOSviewer, CiteSpace, and the Bibliometrix (R package).

**Results:**

A total of 1,815 documents authored by 8,592 researchers from 2,376 organizations across 77 countries and published in 285 journals were identified. The United States led in both publication volume and total citations. The University of Melbourne had the highest number of publications, while Osteoporosis International emerged as the core journal in this field, with the highest number of publications, citations, and H-index. Cawthon PM was the most influential author, with 21 publications and 3,271 citations. Keywords were categorized into four clusters: Cluster 1 (epidemiology and pathophysiology of sarcopenia), Cluster 2 (clinical outcomes), Cluster 3 (management), and Cluster 4 (physical function). The most common keywords were mainly about “sarcopenia”, “body composition”, “muscle strength”, “hip fracture” and “mortality”.

**Conclusions:**

The bibliometric results indicated a steady and rapid increase in the field of sarcopenia and orthopaedic surgery from 2003–2023. Previous research has predominantly focused on the epidemiology, pathophysiology, clinical outcomes, physical function, and management of sarcopenia. Future research in the intersection of sarcopenia and orthopaedic surgery is likely to delve into the molecular mechanisms of muscle-bone crosstalk, and multidisciplinary management of elderly sarcopenic patients in the orthopaedic field.

## 1 Introduction

The concept of sarcopenia was first established by Irwin Rosenberg in 1989 (1). Since then, sarcopenia had garnered increased attention from researches and surgeons. The definition of sarcopenia was formalized in 2010 by the European Working Group on Sarcopenia in Older People (EWGSOP) (2). In 2014, the Asian Working Group on Sarcopenia (AWGS) released the first sarcopenia expert consensus specifically for the Asian population, four years following the European EWGSOP guidelines (3). According to EWGSOP, sarcopenia is a syndrome characterized by low muscle strength, reduced skeletal muscle mass and quantity, and decreased physical activity capability. The definition was updated in 2019, EWGSOP2 (4), which placed much more emphasis on low muscle strength. Sarcopenia might be diagnosed if low muscle strength was detected, and the diagnosed would be confirmed when low muscle mass accompanies low muscle strength. Previous studies had identified risk factors associated with sarcopenia, including oxidative stress (5), mitochondrial dysfunction (6) and nutrition (7) have been identified as the risk factors of sacropenia. A systematic review have confirmed that adding nutritional interventions to exercise had a larger effect on handgrip strength than exercise (8) Sarcopenia represents a progressive and highly prevalent skeletal muscle disorder in aging populations. Currently evidence has demonstrated its substantial impact on functional independence (9), elevated blood transfusion requirements post-total knee arthroplasty (10), diminished quality of life (11), and long-term survival rates (12). Significant associations between muscle and bone size have been documented across the lifespan (13). The term “Muscle-Bone Interactions” indicates not only anatomical relationships but also functional connections between these two systems (14). Currently, there is a growing trend of research on orthopaedic diseases and sarcopenia, including osteoporosis (15), fracture (16), arthroplasty (17). Maria et al. conducted cohort of real-life elderly subjects with musculoskeletal concerns and showed that these subjects were highly susceptible to sarcopenia (18). Exercise is an effective method for preventing and treating sarcopenia, which can improve the skeletal muscle mass, strength, and physical function of patients with sarcopenia to varying degrees (19) However, the relationship between sarcopenia and orthopaedic surgery, as well as the underlying mechanisms is still unclear. Understanding the role of sarcopenia in orthopaedic surgery could potentially assist the clinicians in accelerating patient rehabilitation post orthopadedic surgery.

Bibliometrics, a sub-discipline of library and information science, provides a robust framework for describing and analyzing the dynamics and progress of a specific field. The first bibliometric study, utilizing the top 100 cited articles in the Journal of the American Medical Association, was conducted by E Garfield in 1987 (20).

The aim of this study was to evaluate the global and demestic scientific progress of the sarcopenia-orthopaedic surgery interface. By performing a bibliometric analysis of research outputs, we identified the emerging trends and provided crucial theoretical foundations for future research and clinical protocol development.

## 2 Materials and methods

### 2.1 Data source and search strategy

Web of Science (Wos, Clarivate Analytics, Philadelphia, PA, USA) is highly influential and comprehensive scientific database, extensively utilized in many other bibliometric studies (21, 22).

**Figure 1:**
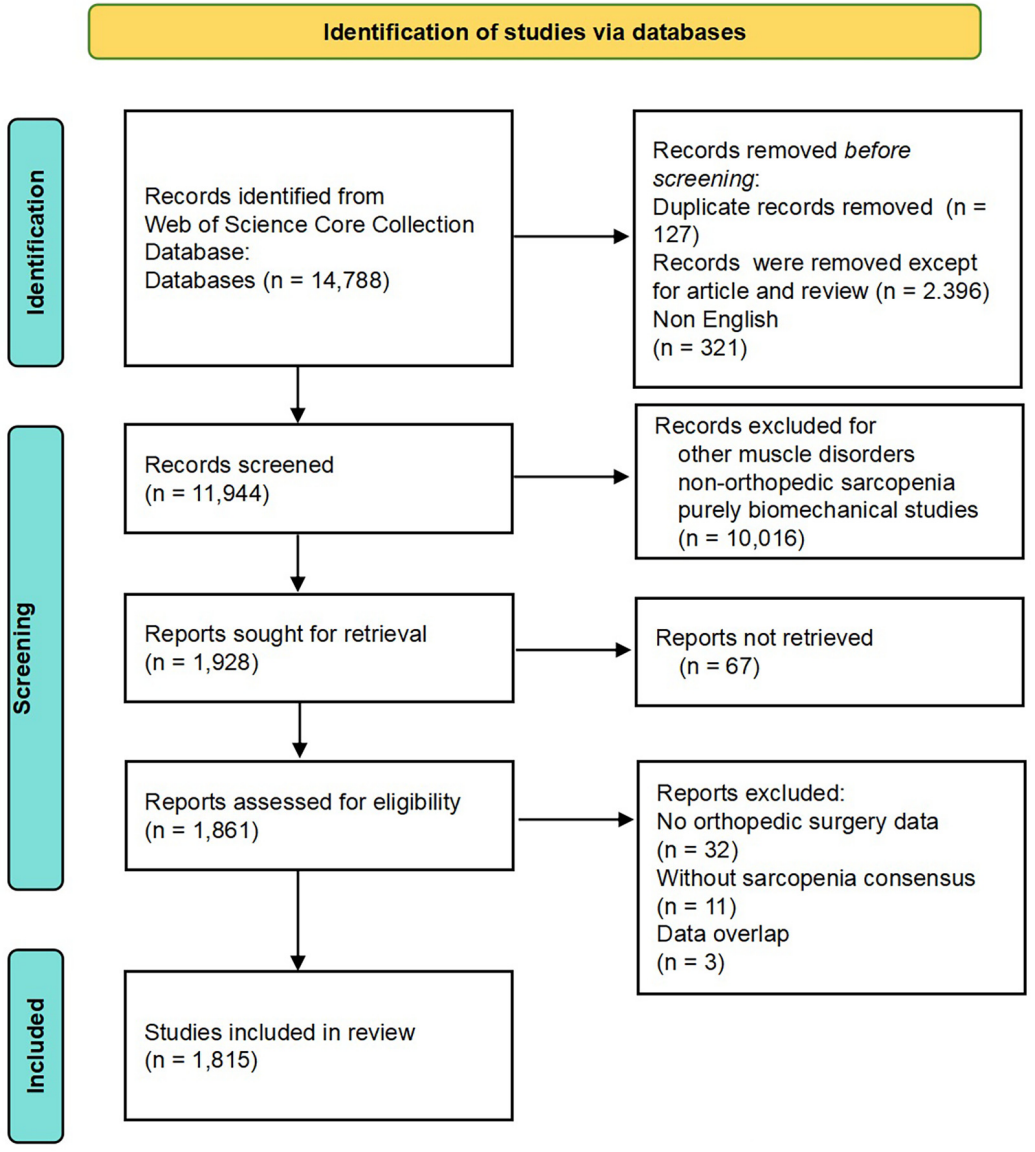
The flowchart of the study.

This study employed the Web of Science Core Collection Database, specifically selecting data indexed in the Science Citation Index Expanded (SCIE) from January 1, 2003, to December 31, 2023. The search strategy for sarcopenia was TS = (“Sarcopenia” OR “Sarcopenias” OR “muscle wasting” OR “Muscular Atroph*” OR “Muscle Atroph*” OR “Sarcopen*” OR “Myope*” OR “Sarcopaen*” OR “Myopaen*”). For the orthopaedic surgery field, the search strategy TS = (“Musculoskeletal Diseases” OR “Orthopedics” OR “Orthopedic Procedures” OR “Musculoskeletal Disease” OR “Orthopedic Disorders” OR “Orthopedic Disorder” OR “Orthopedic Procedure” OR “Procedure, Orthopedic” OR “Procedures, Orthopedic” OR “Orthopedic Surgical Procedures” OR “Orthopedic Surgical Procedure” OR “Procedure, Orthopedic Surgical” OR “Procedures, Orthopedic Surgical” OR “Surgical Procedure, Orthopedic” OR “Surgical Procedures, Orthopedic” OR “Orthopedic Surgery” OR “Orthopedic Surgeries” OR “Surgeries, Orthopedic” OR “Surgery, Orthopedic” OR “Orthopedic Rehabilitation Surgery” OR “Orthopedic Rehabilitation Surgeries” OR “Rehabilitation Surgeries, Orthopedic” OR “Rehabilitation Surgery, Orthopedic” OR “Surgeries, Orthopedic Rehabilitation” OR “Surgery, Orthopedic Rehabilitation” OR “orthopedic*” OR “orthopaedic*” OR “Fracture*” OR “joint dislocation*” OR “Osteo*” OR “Arthro*” OR “Spin*” OR “Vertebr*” OR “Arthroplasty” OR “Joint Replacement”). The final search query combined both search components through Boolean AND. Document types included “articles,” “review articles,” and the language was set to “English”. Only, research articles and reviews were included, while duplicates, other publication types and irrelevant articles were removed. Two researches independently selected and analyzed the documents. After retrieving and screening out the irrelevant references, “full record and cited references” were exported in “Plain Text” format. The detailed literature selection process, including inclusion/exclusion criteria and screening results, is comprehensively illustrated in Figure 1 (PRISMA Flowchart).

### 2.2 Data analysis

For bibliometric analysis and data visualization, this study utilized Microsoft Office Excel 2019, CiteSpace 6.1.R3, VOSviewer 1.6.18, R, Bibliometrix were used to employ the overall descriptive analysis.

Quantitative and qualitative analyses were conducted by VOSviewer including: the co-occurrence of authors, countries, institutions, journals, keywords, references and co-cited analyses of authors, references. CiteSpace was used to analyze the burst strength of the keywords and conferences. Microsoft Office Excel 2019 was used to analyze the annual publication numbers. Additionally, Bibliometrix (R package) or online bibliometric platform (https://bibliometric.com/) were also used to visualize the collaboration of authors, countries, analyse the H-index of authors and journals.

The visual maps generated in this study consist of nodes and links. Nodes represent different research factors such as authors, journals, countries, or keywords. The size of the nodes denotes the frequency of publications, citations, or occurrences. The links between nodes reflect the relationships between them, with thicker lines indicating stronger associations.

## 3 Results

### 3.1 Analysis of research trends

**Figure 2:**
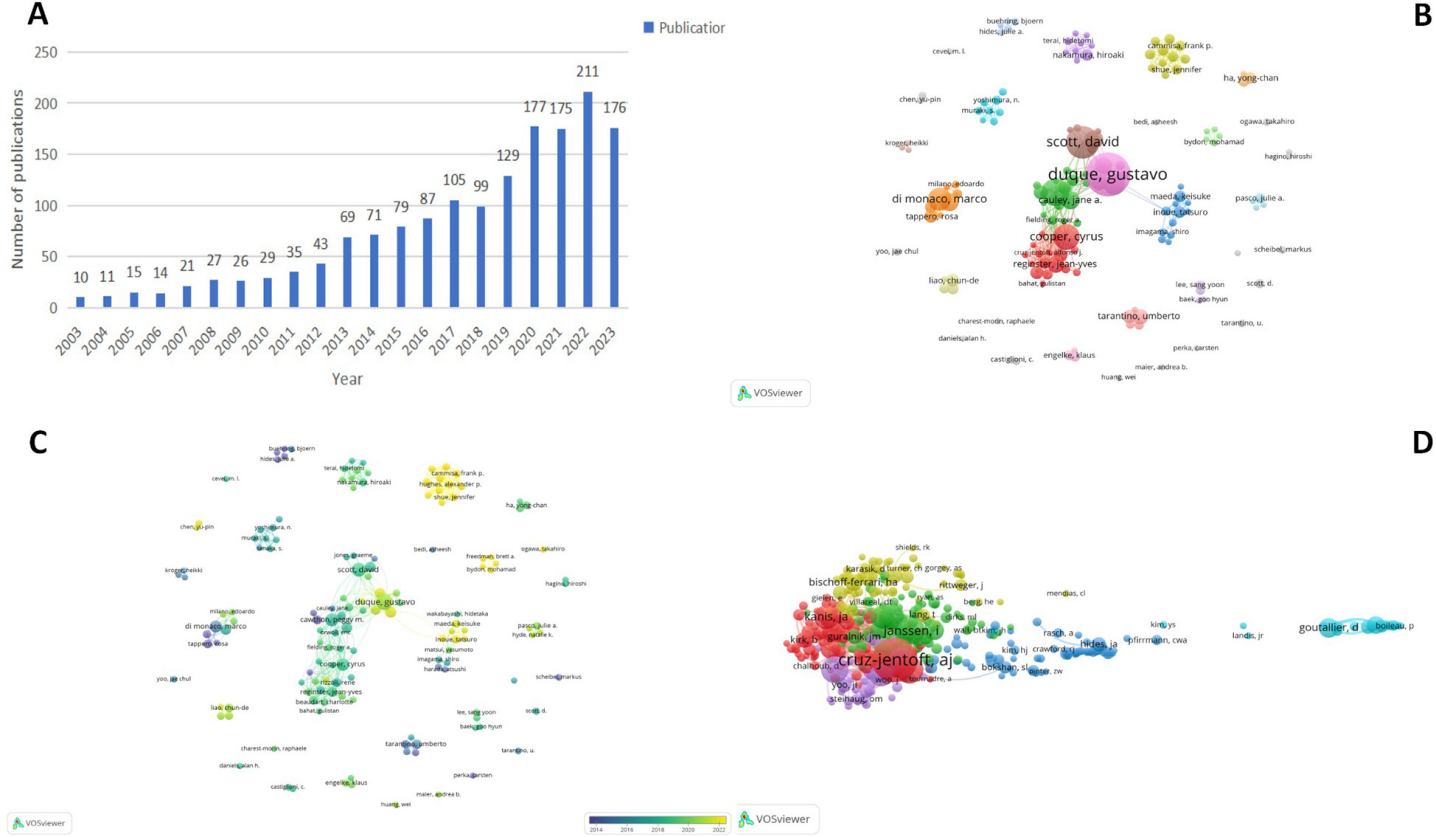
Descriptive analysis of the annual publications and authors. **(A)** The annual number of publications on the topic of sarcopenia and orthopaedic surgery research from 2003–2023. **(B)** Network mapping of the collaborated authors. **(C)** Overlay mapping of the collaborated authors. **(D)** Network mapping of the co-cited authors.

From 2003–2023, a total of 39,611 publications related to sarcopenia and 1,740,304 publications related to orthopaedic surgery were retrieved from the WOS database, 1,815 documents specifically addressing sarcopenia within the context of orthopaedic surgery field were included in the present study, comprising 1,457 original research articles and 358 review articles. These articles were authored by 8,592 researches from 2,376 institutions in 77 countries and were published in 285 journals. Figure 2 enlists the descriptive analysis of the annual publications and author, furthermore, Figure 2A illustrates the time trend of publications for sarcopenia in orthopaedic surgery field, showing a consistent increase over time. A slow publication rate was observed before 2013. However, post 2013, there has been a continuous rise in publications. Rising from 69 in 2013–211 in 2022, an approximately 300% increase. Since 2018, the annual publication number has consistently exceed 100. In 2022, the publication volume peaked at 211 papers. The number of publications in 2022 (211) was nearly 21.1-fold increase comparing to 2003 (10). This growth indicates a growing global interest in sarcopenia and orthopaedic surgery, signaling this area has become a significant research hotspots.

### 3.2 Analysis of authors and co-cited authors

Table 1 lists the citation metrics, number of publications, and H-index values for the top 10 authors specializing in sarcopenia and orthopaedic surgery.

**Table 1:** The general information of top 10 authors in sarcopenia and orthopaedic surgery between 2003 and 2023.

Rank	Author	Publications	Citations	Average Citation/Publication	H-index
1	Duque, Gustavo	34	975	28.68	19
2	Scott, David	25	739	29.56	15
3	Cawthon, Peggy M.	21	3,271	155.76	16
4	Copper, Cyrus	20	1,869	93.45	21
5	Di monaco, Marco	18	547	30.39	12
6	Krik, Ben	16	497	31.06	11
7	Cauley, Jane	14	1,130	80.71	11
8	Ebeling, Peter	14	458	32.71	11
9	Castiglioni, Carlotta	14	278	19.86	8
10	Reginster, Jean Yves	13	1,595	122.69	15

These 10 authors published a total of 189 articles, accounting for 10.4% of all included articles. Duque, Gustavo is the most productive author in the field with the highest number of publications (*n* = 34). The H-index reflects the number and quality of authors' publications. Precisely, a writer has published H papers, each of which has been cited at least H times by the other articles (23). The top three H-index is Copper, Cyrus (21); Duque, Gustavo (19) and Cawthon, Peggy M (16). The number of top three authors' citation number are more than 1,500 times: Cawthon Peggy M (citation = 3,271), Copper Cyrus (citation = 1,869), and Reginster Jean Yves (citation = 1,595), highlighting the significant impact of their works. Furthermore, the collaborative relationships can enhance the productivity within a certain field. Figure 2B visualize the collaborated relation among the included authors. The same color represents an author cluster, which means these authors have tight cooperation with each other. The thicker of the line, the closer of the collaboration. The node size of Cawthon Peggy M. and Cauley Jane A. are larger due to much more publications compared to others. Besides that, the close collaboration is also observed among several authors, such as Scott David, Duque Gustavo, Reginester Jean, etc. Figure 2C displays an overlay map of co-cited authors. The color of the nodes represents the average publication year of the including articles. Cammisa and Girardi et al. make significant contribution to the field in recent years.

Figure 2D shows a co-cited authors map. The most prominent nodes are linked to influential authors who have established the foundation work in sarcopenia and orthopaedic surgery. The top three co-cited authors are Cruz-Jentoft AJ (*n* = 874), and Janssen I (*n* = 327), Chen Ik (*n* = 325).

### 3.3 Analysis of countries or regions

The productivity of countries with more than ten publications is visualized using VOSviewer. The top ten productive countries are shown in Table 2. The leading countries in terms of publications on sarcopenia in orthopaedic surgery field are the United States, Japan, China, South Korean, and Australia, with the United States significantly ahead in both publication volume (*n* = 404) and citation counts (*n* = 20,240). Notably, publication number of China, Japan and South Korea have surged since 2016, reflecting the impact of aging populations and increased research focus in East Asia.

**Table 2:** Top ten countries in sarcopenia and orthopaedic surgery field.

Rank	Country	Publications	Citations	Average Citations/Publication
1	The United States	404	20,240	50.09
2	Japan	201	5,669	28.2
3	China	191	3,483	18.24
4	South Korean	179	3,724	20.8
5	Australia	166	7,559	45.54
6	Germany	135	4,197	31.09
7	Italy	124	6,095	48.86
8	England	117	7,026	39.69
9	Canada	68	3,593	52.84
10	France	65	4,674	71.91

**Figure 3:**
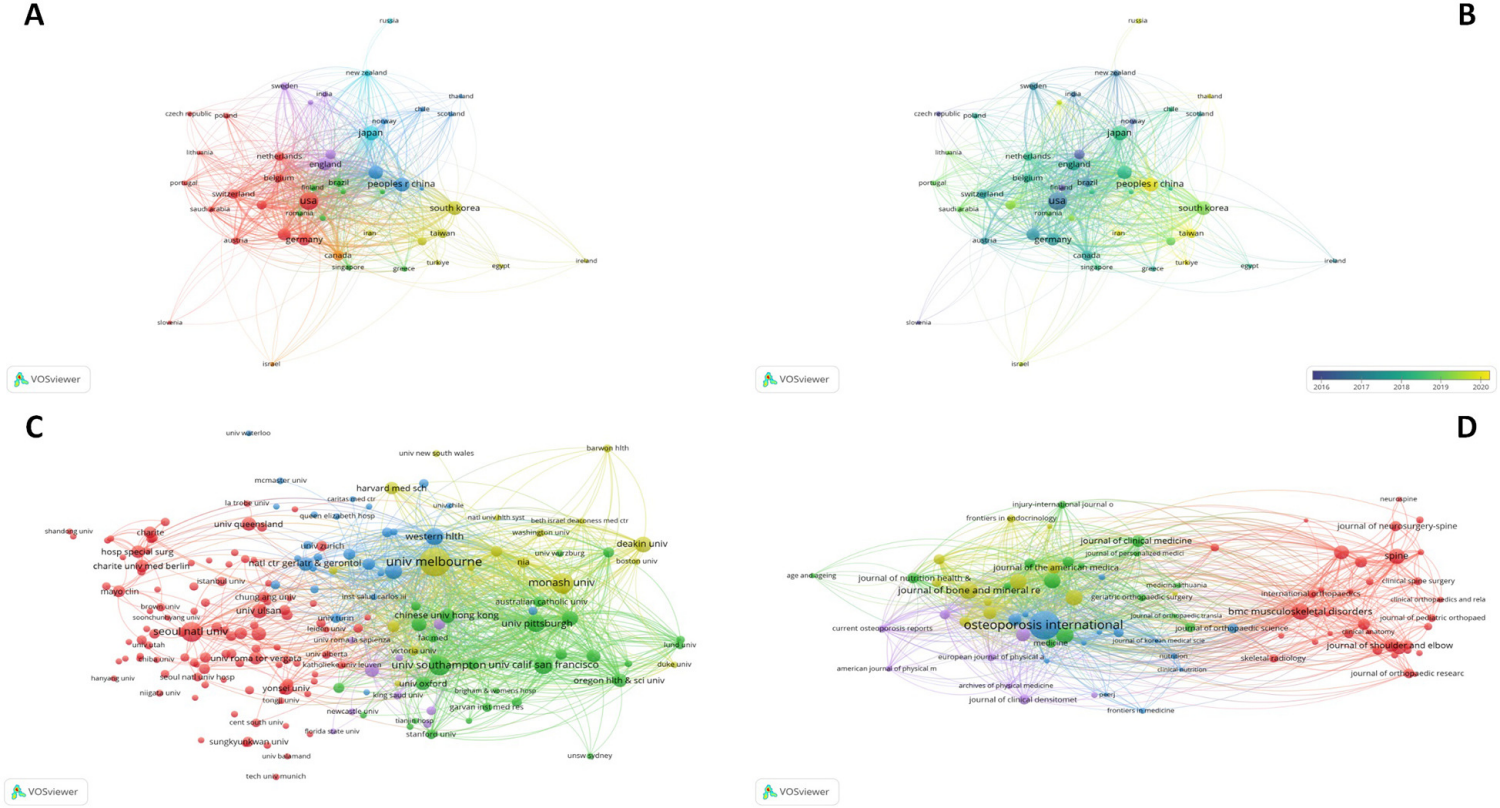
Descriptive analysis of the collaborated countries, institutions and the citation relationships between the journals. **(A)** Network mapping of the collaborated countries. **(B)** Overlay mapping of the collaborated countries. **(C)** Network mapping of the collaborated institutions. **(D)** Network mapping of the citation relationships between the journals.

Figure 3A depicts the global collaboration among these countries. Node size is proportional to the number of publications, and the line thickness represents citation frequency. It's obvious that the distribution of publications is notably uneven, with a significant top-heavy effect, where the majority of articles coming from a few countries. The United States, China, Japan and Germany show the most frequent citation relation. Figure 3B shows an overlay map of country collaborations, with yellow color indicating recent publications, suggesting rapid research development in China.

### 3.4 Analysis of institutions

A total of 909 institutions have researched the sarcopenia and orthopaedic surgery. The top ten institutions with the largest number of publications are listed in Table 3. These institutions are located in four countries: Australia (four institutions), The United States (three institutions) and South Korea (one institution) and England (one institution). The University of Melbourne is the most productive institution in this field, with over 76 publications.

**Table 3:** The top 10 institutions in sarcopenia and orthopaedic surgery field.

Rank	Institution	Country/Region	Publications	Citations	Average Citation/Publication
1	The University of Melbourne	Australia	76	2,721	35.8
2	Monash University	Australia	42	999	23.79
3	Seoul National University	South Korea	40	1,134	28.35
4	University of Southampton	England	39	3,175	81.41
5	University of California, San Francisco	The United States	33	1,632	49.45
6	University of Pittsburgh	The United States	32	4,297	134.28
7	Western health	The United States	31	1,174	37.87
8	Deakin University	Australia	27	483	17.89
9	The University of Sydney	Australia	27	1,439	53.3
10	California Pacific Medical Center	The United States	25	3,811	152.44

Figure 3C illustrates the co-institution network with 2,376 nodes and 43,713 links, showing close collaborative relationships, particularly between the University of Melbourne, Monash University and Seoul National University. These institutions make a closely association and play a significant part to the field.

### 3.5 Analysis of journals and co-cited journals

A total of 285 journals have published research focusing on sarcopenia and orthopaedic surgery. Among these, 30 journals had at least 18 publications. The top ten productive journals in this field are shown in the Table 4. Figure 3D highlights that Osteoporosis International have the highest number of publications (*n* = 125) and citations (*n* = 2,621), focusing primarily on the detection, prevention and management of sacropenia and osteoporosis. The impact factors for the top 10 cited articles were obtained in 2024.

**Table 4:** General information of top 10 journals in the sarcopenia and orthopaedic surgery.

Rank	Source	Publications	Citations	Average Citation/Publication	H-index	Impact factor
1	Osteoporosis International	125	2,621	20.97	29	3.86
2	Journal of Bone and Mineral Research	50	1,832	36.64	20	6.2
3	BMC Musculoskeletal Disorders	46	854	18.57	17	2.56
4	Aging Clinical and Experimental Research	46	648	14.09	15	3.97
5	Journal of Cachexia Sarcopenia and Muscle	45	2,408	53.51	20	12.51
6	Spine	43	1,619	37.65	19	4.17
7	Calcified Tissue International	41	1,482	36.15	19	3.95
8	Nutrients	41	1,048	25.56	17	5.72
9	European Spine Journal	35	1,074	30.69	18	2.8
10	Journal of Shoulder and Elbow Surgery	34	623	18.32	15	3.02

**Figure 4:**
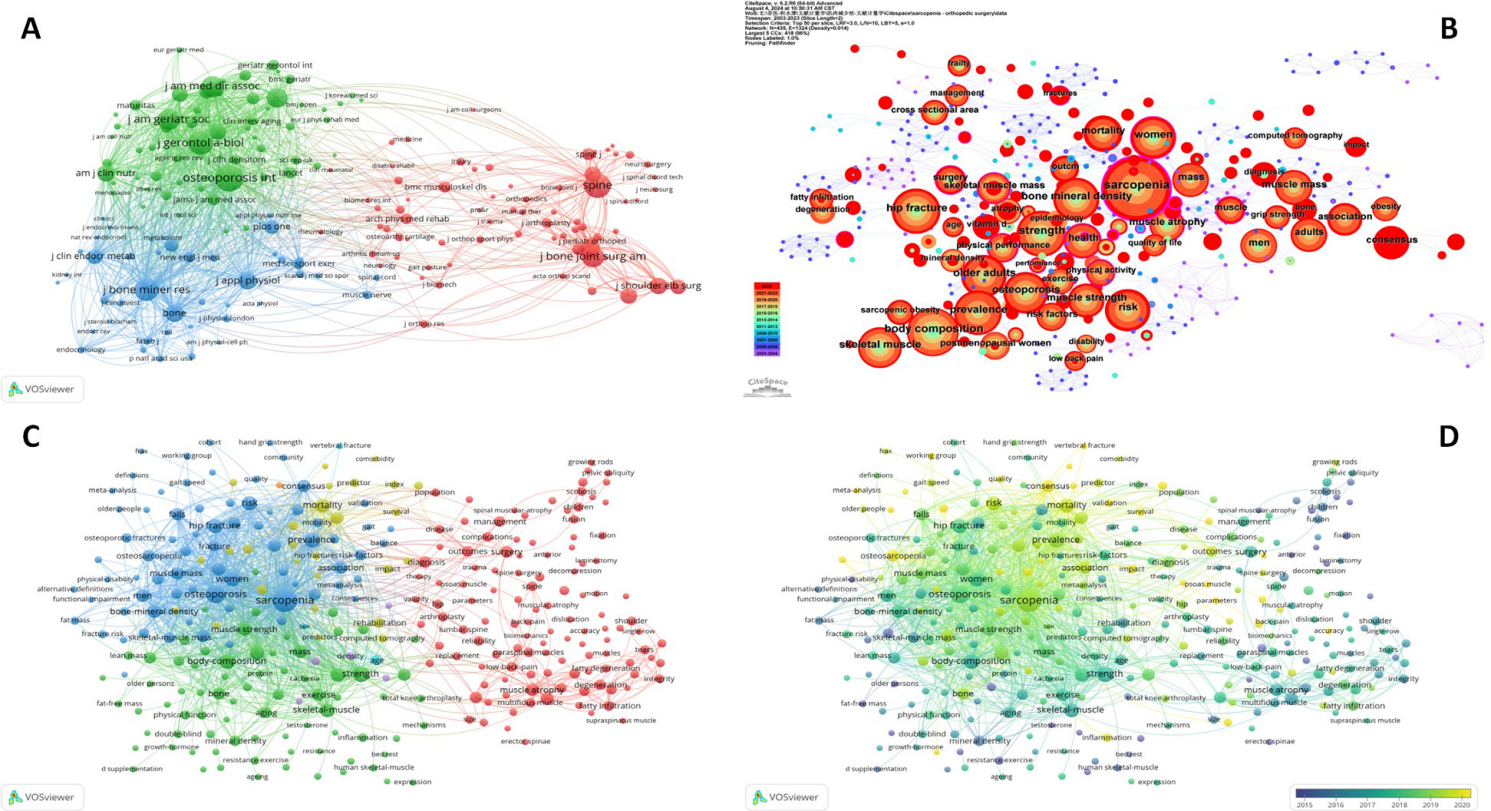
Descriptive analysis of the co-cited journals and co-occurrence keywords. **(A)** Network mapping of the co-cited journals. **(B)** Tree-ring mapping of the co-occurrence keywords. **(C)** Network mapping of the co-occurrence keywords. **(D)** Overlay mapping of the co-occurrence keywords.

Figure 4A demonstrates the co-cited journals in this field. The most frequently co-cited journal is Osteoporosis International. The top 5 journals with the highest total link strength are Journal of Bone And Mineral Research (IF = 6.44), Osteoporosis International (IF = 3.59), Gerontology Series A: Biological Sciences And Medical Sciences (IF = 5.10), American Geriatrics Society (IF = 6.30), Bone (IF = 4.15). The Journal of Bone And Mineral Research has the strongest co-cited relationship with Osteoporosis International. Three of the top five journals are related to sarcopenia and osteoporosis, which means that there is a tight relationship between sarcopenia and osteoporosis. In the orthopaedic field, the Journal of Spine, Bone And Joint Surgery-American Volume are prominent.

### 3.6 Co-occurrence analysis of keywords

Keywords encapsulate the core content and essence of academic articles. A co-occurrence analysis of keywords can elucidate the prevailing research hotspots within a specific field. Utilizing CiteSpace, we selected 4,985 keywords from the 1,815 publications in this field. As depicts in Figure 4B, the node size represents the frequency of occurrences. Different color means different occurrence time, the yellow relates to the most recent publication, while the blue relates to earlier publiaction years. The most frequent keyword in the researches is “sarcopenia” (*n* = 825 times).

Figure 4C depicts the co-occurrence keywords visualized by VOSviewer, which can be categorized into four distinct clusters, each cluster representing a specific research direction. Nodes within each cluster indicate related research themes. The blue cluster delineates sarcopenia epidemiology and pathophysiology; yellow focuses on clinical outcomes; red concentrates on therapeutic management of sarcopenic patients; green specifically analyzes physical function assessment. Figure 4D provides an overlay visualization of the co-occurrence keywords, with brighter color (yellow), indicates more recent research trends. Research focus has transitioned toward osteosarcopenia, consensus guidelines, and meta-analytical approaches. Emerging themes prominently feature “elderly/aging”, “frailty”, and “muscle strength” (handgrip quantification), while “osteoporosis/bone density metrics”, “fragility fracture epidemiology”, and “survival prediction models” demonstrate accelerated development. Investigation priorities for “osteoarthritis pathogenesis” and “spinal pathology correlations” have become less prominent.

**Figure 5:**
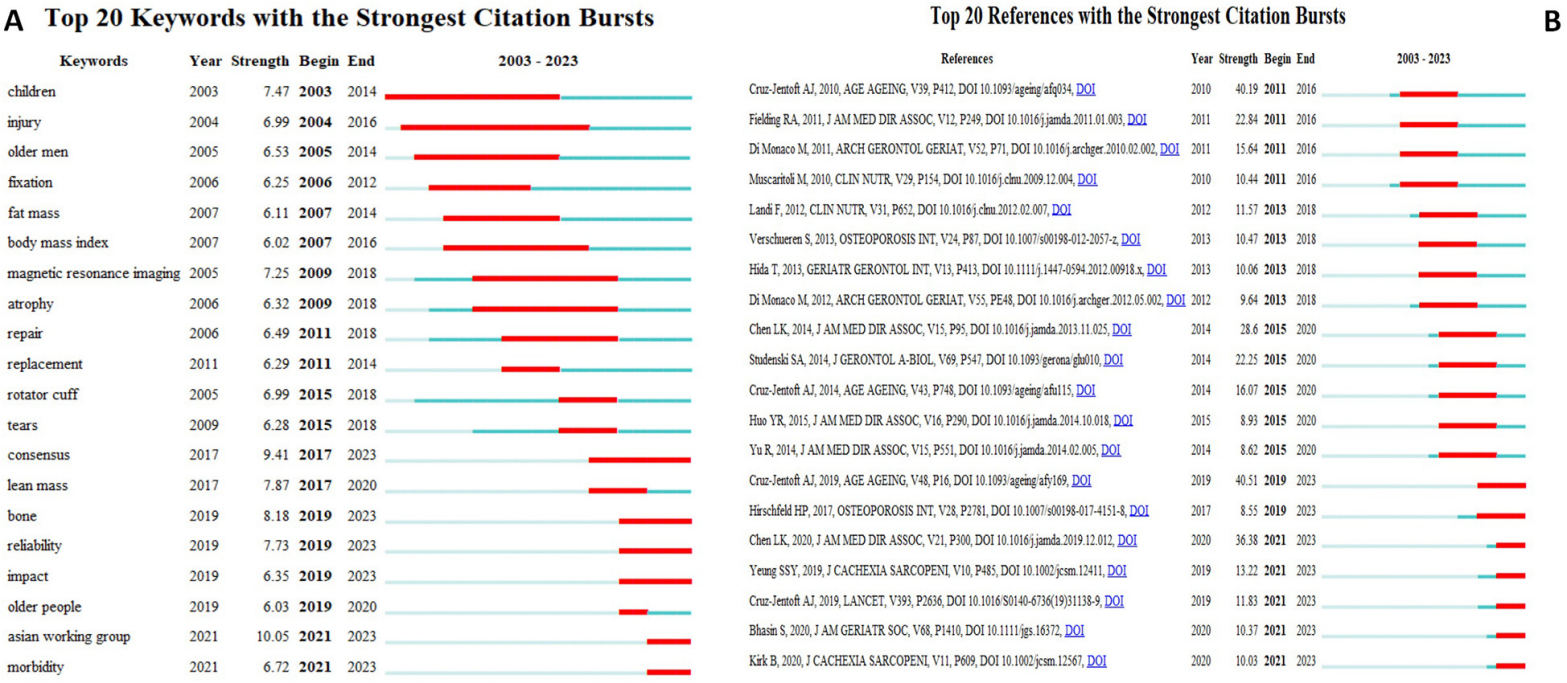
The top 20 keywords, references with the strongest citation bursts. **(A)** The top 20 keywords with the strongest citation bursts between 2003 and 2023. **(B)** The top 20 references with the strongest citation bursts between 2003 and 2023.

To further elucidate emerging research hotspots in sarcopenia, a burst keyword analysis was performed using CiteSpace. Figure 5 shows the top 20 keywords, references with the strongest citation bursts. The red lines in Figure 5A indicate periods of significant keyword bursts, while blue lines represent time intervals. The analysis revealed that, from 2003–2017, research primarily focuses on “injury”, “fixation”, and “BMI”. Since 2017, the focus has shifted towards “consensus”, “treatment strategies”, and “clinical outcomes” related to sarcopenia.

### 3.7 Analysis of highly-cited references

The citation network was analyzed using VOSviewer, which identified 1,815 nodes and 5,916 links. Among the documents cited more than 400 times, the top 10 most-cited articles are listed in Table 5. Notably, half of these articles were published in journals specializing in geriatrics, underscoring the strong association between sarcopenia and the elderly population. The article “The FNIH Sarcopenia Project: Rationale, Study Description, Conference Recommendations, and Final Estimates,” published in the Journals of Gerontology Series A: Biological Sciences and Medical Sciences, received the highest citation count with 1,492 citations.

**Table 5:** The top 10 highly-cited references in sarcopenia and orthopaedic surgery between 2003 and 2023.

Rank	Title	Journal	First Author	Publication Year	Citations
1	The FNIH sarcopenia project: rationale, study description, conference recommendations, and final estimates	Journals of Gerontology Series A-Biological Sciences and Medical Sciences	Studenski, Stephanie A	2014	1,492
2	The healthcare costs of sarcopenia in the United States	Journal of American Geriatrics Society	Janssen, I	2004	972
3	Epidemiology and Burden of Osteoarthritis	British Medical Bulletin	Litwic, Anna	2013	750
4	Sarcopenia in daily practice: assessment and management	BMC Geriatrics	Beaudart, Charlotte	2017	699
5	SARC-F: A simple questionaire to rapidly diagnose sarcopenia	Journal of The American Medical Directors Association	Malmstrom, Theodore K	2013	578
6	Frality and Risk of falls, fracture, and mortality in older women: the study of osteoporotic fracture	Journals of Gerontology Series A-Biological Sciences and Medical	Ensrud, Kristine E	2007	517
7	Clinical results of arthroscopic superior capsule reconstruction for irreparable rotator cuff tears	Arthroscopy-The Journal of Arthroscopic and Related Surgery	Mihata, Teruhisa	2013	508
8	Sarcopenia and its association with falls and fractures in older adults: a systematic review and meta-analysis	Journal of Cachexia Sarcopenia and Muscle	Yeung, Suey S	2019	506
9	Epidemiology and social costs of hip fracture	Injury-International Journal of The Care of The Injured	Verinese, Nicola	2018	470
10	Pitfalls in the measurement of muscle mass: a need for a reference standard	Journal of Cachexia Sarcopenia and Muscle	Buckinx, Fanny	2018	457

References with citation bursts are those that have experienced a significant increase in citations over a specific period, indicating heightened interest and relevance (24). Figure 5B presents the top 20 references with the strongest citation bursts. The earliest burst (strength = 40.19) occurred in the study entitled “Sarcopenia European consensus on definition and diagnosis Report of the European Working Group on Sarcopenia in Older People” published on Age Ageing by Cruz-Jentoft in 2011 (2), which summarized current available data defining sacopenia cut-off points by age and gender. The strongest burst (strength = 40.51) occurred in the study entitled “Sarcopenia: revised European consensus on definition and diagnoses” published on Age Aging by Cruz-Jentoft in 2019, which aimed to enhance awareness of sarcopenia and its risk according to the updated recommendations in EWGSOP2 (4). Additionally, seven references are currently exhibiting bursts (4, 25–30).

## 4 Discussion

As an independent and newborn technology, bibliometrics enables the analysis of extensive publication datasets to uncover emerging research trends and hotspots of interest. In this study, the Science Citation Index Expanded (SCIE) database was utilized to gather data on publications related to sarcopenia and orthopaedic surgery. Various bibliometric visualization tools, including VOSviewer, CiteSpace, Bibliometrix, and Microsoft Excel, were employed to analyze the selected articles. The analysis identified 1,815 articles authored by 8,592 researchers from 2,376 institutions across 77 countries and published in 285 journals. Descriptive statistics indicated a steady increase in the number of publications over the past two decades.

Author Cawthon, Peggy M has made great efforts to research on sarcopenia and orthopaedic surgery. The most frequently cited article (*n* = 1,523) entitled “The FNIH Sarcopenia Project: Rationale, Study Description, Conference Recommendations, and Final Estimates” was published on Journal of Gerontology Series A-Biological Sciences and Medical Sciences by Cawthon, Peggy M in 2014 (31). The Foundation for the National Institutes of Health Biomarkers Consortium Sarcopenia Project (FNIH Sarcopenia Project) conducted an evidence-based approach to establish diagnostic criteria for sarcopenia, such as grip strength thresholds (<26 kg for men and <16 kg for women). Overlay visualizations of co-occurrence keywords reveal that consensus on sarcopenia is emerging as a prominent research focus. Achieving a consensus on defining sarcopenia as a clinical condition remains a critical issue.

The analysis of co-cited references helps to map the knowledge base in the field (32). In the top ten highly cited references in sarcopenia and orthopaedic surgery field, six of them mainly focus on the consensus, diagnosis, clinical outcomes and managements of sacropenia (29, 31, 33–36). Furthermore, seven articles with the strongest citation bursts continue to exhibit notable influence (4, 25–30). These articles concentrate on the definition (4, 30), prevention (28), clinical outcomes (30) of sarcopenia and the epidemiology, diagnosis, and treatments of osteosarcopenia (26, 27). The references highlights the sarcopenia of orthopaedic surgery from diverse perspectives. Obviously, the clinical outcomes and prevention of orthopaedic patients with sarcopenia has become another research hotspots.

The co-occurrence keywords visualized by VOSviewer can be roughly classified into 4 clusters. Each cluster represents a distinct academic direction, including cluster 1 (epidemiology and pathophysiology of sarcopenia), cluster 2 (clinical outcomes), cluster 3 (management), and cluster 4 (physical function).

Yuan et al. estimated that sarcopenia affects 10%–16% of the elderly population globally, with higher prevalence among patients compared to general populations. Physical inactivity, malnutrition and diabetes have been identified as the risk factors of sarcopenia (37). Nishikawa et al. further elucidated the mechanisms of primary sarcopenia, including insulin resistance, oxidative stress, malnutrition and hormonal changes of elderly patients (38). Elucidating sarcopenia's epidemiological patterns and pathophysiological mechanisms is pivotal for designing evidence-based therapeutic interventions.

Emerging clinical evidence consistently demonstrates the significant impact of sarcopenia on orthopedic surgical outcomes. A 3.51-fold increased odds of knee osteoarthritis development compared to non-sarcopenic controls (OR = 3.51) have been identified (39). Similarly, sarcopenia is associated with the higher prevalence of multiple osteoporotic vertebral fractures in women (OR = 2.56) (40). He et al. conducted a case-control retrospective cohort study, which found that sarcopenic patients undergoing the total knee arthroplasty had lower postoperative functional scores and higher complication rates (41). Moreover, severe sarcopenia demonstrates significant negative associations with key postoperative metrics following the total hip arthroplasty, manifesting as delayed functional recovery, reduced hip function scores and lower patients-reported outcomes at 6 months after surgery (42). Sarcopenia is closely related to the occurrence of orthopedic diseases and the rehabilitation of orthopedic surgery, and early intervention for patients with sarcopenia will help improve the prognosis and functional recovery of such patients.

Current evidence-based management strategies emphasize the exercise interventions and nutritional optimization. The management of sarcopenia primarily concentrates on the progressing resistance training for muscle strengthening and gait training. Moreover, multimodal therapy would also incorporate improvement in the grip strength and extremity skeletal muscle mass elderly patients with sarcopenia (43, 44). Dietary protein and physical activity have already recognized as the key stimuli for muscle protein synthesis. Sufficient intakes of protein, vitamin D and antioxidant nutrients are conducive to alleviate the severity of sarcopenia. However, evidence form these articles is observational and from high-income countries (45). Expert guidelines support that the time of nutritional intake before and after exercise is critical to the increase muscle mass and benefit to sarcopenia (46). Combined nutrition supplementation and physical exercise could improve muscle mass, strength and fat mass among sarcopenic elderly (47). Future researches need to pay more attention to these areas and figure out whether there is a relevant dose-response relationship.

In terms of physical function of the sacopenic patients. Sarcopenia is associated with the lower physical function, such as lower muscle strength and slower walking ability, addressing muscle weakness and enhancing physical activity may benefit sarcopenic patients. Hand grip strength (HGS) has been identified as an important biomarker of health (48). A cross-sectional study conducted by Zeng et al. indicated that females were much more likely to experience significantly lower hand grip strength (HGS) and gait speed (GS) values, unstructured daily routine was associated with the risk of low GS in older Chinese. Measures of GS, HGS could provide a readily available and effective method for assess the risk of sarcopenia (48, 49). HGS could independently reveal changes in nutritional status (50), which responds earlier than other measurements to nutritional deprivation (2). For example, appendicular muscle mass and grip strength are significantly higher in patients treated with nutrition supplementation (e.g., whey protein, fish oil, and vitamin D) and fat mass is significantly lower in nutrition groups (47).

## 5 Strengths and limitations

To the best of our knowledge, this study is the first bibliometric analysis to include classical literature related to sarcopenia and orthopedic surgery. A comprehensive literature review has been conducted to enhance the accuracy and validity of the findings. However, there are limitations. Firstly, only publications indexed in SCIE were included, excluding articles not covered by this database. Secondly, the study focused exclusively on English-language research and review articles, potentially missing relevant non-English literature. However, these limitations are unlikely to substantially compromise the validity or reliability of our bibliometric analysis and its primary findings. According to the temporal range, the research published in 2024 may have been overlooked due to its recent publication. Thirdly, we acknowledge that due to technical limitations in the export format, our analysis could not completely exclude self-citations. This limitation may affect the generalizability of our findings and should be considered when interpreting the results.

## 6 Conclusion

Our study provided a comprehensive landscape of the publication development and essential points of sarcopenia in orthopedic surgery over the past 20 years. Research on sarcopenia and orthopaedic surgery has demonstrated a stable and rapid increase. Key research areas have encompassed epidemiology, clinical outcomes, management strategies, and physical function. However, current diagnostic standards exhibit slight discrepancies due to variations in diagnostic tools and populations. Exercise and nutritional interventions have proven to be the most effective treatments, while pharmacological approaches remain under investigation. Moreover, future trends in the cross field of sarcopenia and orthopaedice would be the molecular mechanisms of crosstalk between muscles and bones, multidisciplinary management.

## Data Availability

The original contributions presented in the study are included in the article/Supplementary Material, further inquiries can be directed to the corresponding authors.
